# PD-1 alters T-cell metabolic reprogramming by inhibiting glycolysis and promoting lipolysis and fatty acid oxidation

**DOI:** 10.1038/ncomms7692

**Published:** 2015-03-26

**Authors:** Nikolaos Patsoukis, Kankana Bardhan, Pranam Chatterjee, Duygu Sari, Bianling Liu, Lauren N. Bell, Edward D. Karoly, Gordon J. Freeman, Victoria Petkova, Pankaj Seth, Lequn Li, Vassiliki A. Boussiotis

**Affiliations:** 1 Division of Hematology-Oncology, Harvard Medical School, 330 Brookline Avenue, Boston, Massachusetts 02215, USA; 2 Department of Medicine, Beth Israel Deaconess Medical Center, Harvard Medical School, 330 Brookline Avenue, Boston, Massachusetts 02215, USA; 3 Beth Israel Deaconess Cancer Center, Harvard Medical School, 330 Brookline Avenue, Boston, Massachusetts 02215, USA; 4 Metabolon, Inc., 617 Davis Drive, Suite 400, Durham, North Carolina 27713, USA; 5 Division of Medical Oncology, Dana-Farber Cancer Institute, Boston, Massachusetts 02284-9168, USA; 6 Division of Interdisciplinary Medicine and Biotechnology, Beth Israel Deaconess Medical Centerr, Harvard Medical School, 330 Brookline Avenue, Dana 513-517, Boston, Massachusetts 02215, USA

## Abstract

During activation, T cells undergo metabolic reprogramming, which imprints distinct functional fates. We determined that on PD-1 ligation, activated T cells are unable to engage in glycolysis or amino acid metabolism but have an increased rate of fatty acid β-oxidation (FAO). PD-1 promotes FAO of endogenous lipids by increasing expression of CPT1A, and inducing lipolysis as indicated by elevation of the lipase ATGL, the lipolysis marker glycerol and release of fatty acids. Conversely, CTLA-4 inhibits glycolysis without augmenting FAO, suggesting that CTLA-4 sustains the metabolic profile of non-activated cells. Because T cells utilize glycolysis during differentiation to effectors, our findings reveal a metabolic mechanism responsible for PD-1-mediated blockade of T-effector cell differentiation. The enhancement of FAO provides a mechanistic explanation for the longevity of T cells receiving PD-1 signals in patients with chronic infections and cancer, and for their capacity to be reinvigorated by PD-1 blockade.

## Abstract (web summary)

Activation of T cells results in metabolic reprogramming to favour glycolysis. Here, Patsoukis *et al*. show that the surface receptor PD-1 inhibits glycolysis and increases the metabolism of lipids, providing a potential mechanism for the blockade of T effector functions but also for the longevity accompanying T cell exhaustion.

## Graphical abstract

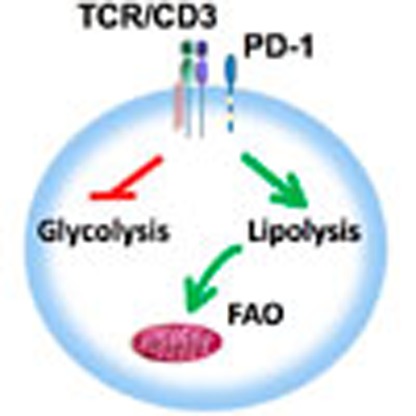

Maintenance of peripheral tolerance is essential for homeostasis of the immune system. While central tolerance mechanisms result in deletion of the majority of self-reactive T cells, some T lymphocytes specific for self-antigens escape this process and circulate in the periphery. PD-1 (CD279) and its ligands, PD-L1 (B7-H1; CD274) and PD-L2 (B7-DC; CD273), play a vital role in peripheral tolerance^1^. PD-1 exerts its effects during the initial phase of activation of autoreactive T cells on self-antigen presentation by DC. In addition, PD-L1/2, expressed on non-haematopoietic tissues, mediate tissue tolerance by suppressing tissue-reactive T cells^2^,^3^. In contrast to its beneficial role in maintaining self-tolerance, PD-L1/2 expressed on malignant tumours or tumour-infiltrating myeloid cells, mediate potent inhibitory signals on effector T cells and have detrimental effects on anti-tumour immunity^4^,^5^. Moreover, expression of PD-1 by ‘exhausted’ T cells in chronic viral infections prevents the function of virus-specific T-cell effectors and viral clearance^6^,^7^.

Naïve T cells utilize oxidative phosphorylation (OXPHOS) for energy generation. On activation via the T cell receptor, T cells undergo a metabolic reprogramming to glycolysis, which is required to support their growth, effector differentiation and function^8^,^9^. Although energetically less efficient, glycolysis is required for cell growth. Conventional views suggest that proliferating cells have a high rate of aerobic glycolysis, even though there is sufficient oxygen present to support OXPHOS, a phenomenon known as the Warburg effect^10^. Signals from the CD28 co-stimulatory pathway and the γ-chain signalling cytokines support activation, growth and expansion of T cells by promoting this metabolic programme^11^,^12^. Divergence in the metabolic reprogramming is critical to effectively imprint distinct T-cell fates. This has been shown with the switch to glycolysis that accompanies effector T-cell differentiation^13^ and the switch to fatty acid β-oxidation (FAO) that accompanies the conversion of T-effector to T-memory cells 14. Furthermore, enforcing FAO by elevating AMPK activity or by inhibiting the mammalian target of rapamycin resulted in increased numbers of memory T cells^14^,^15^. It remains unknown whether the functional outcome of PD-1 ligation is linked to T-cell reprogramming to a specific metabolic pathway.

We investigated the metabolism of T cells receiving PD-1 signals and discovered that they were unable to engage in glycolysis, glutaminolysis or metabolism of branched-chain amino acids but displayed an increased rate of FAO. PD-1 promoted FAO of endogenous lipids by increasing the rate-limiting enzyme of FAO, carnitine palmitoyl transferase (CPT1A) and inducing lipolysis as determined by the increase of the major triacylglycerol (TG) hydrolase desnutrin/adiposite triglyceride lipase (ATGL) and release of fatty acids and glycerol. In addition to increased FAO, T cells stimulated in the presence of PD-1 ligation possessed substantial spare respiratory capacity (SRC), the extra mitochondrial capacity available in the cell to produce energy under conditions of stress. Because burning fat has a strong association with longevity in many cell types^16^,^17^,^18^, these unexpected findings indicate that PD-1 ligation enables T cells to survive as long-lived cells by utilizing a fat-based metabolism. In contrast, CTLA-4 inhibited expression of the glutamine transporters SNAT1 and SNAT2, and the glucose transporter Glut1 and inhibited glycolysis without augmenting CPT1A and FAO, suggesting that CTLA-4 maintains immune quiescence by preserving the metabolic profile of non-stimulated cells. PD-1 also altered the metabolic programme of pre-activated CD4^+^ T cells and reprogrammed their metabolism from glycolysis to FAO. Because PD-1 is expressed on activated T cells, which employ glycolysis as the dominant source of energy generation during differentiation into effectors, our findings indicate that PD-1 ligation prevents effector cell development by altering metabolic reprogramming of activated T cells by inhibiting glycolysis and promoting FAO. Furthermore, the enhancement of FAO suggests a mechanism responsible for the survival and persistence of T cells receiving PD-1 signals in patients with chronic infections and cancer and for their capacity to be reinvigorated by PD-1 blockade.

## Results

### PD-1 abrogates transport and utilization of glucose

**Figure 1:**
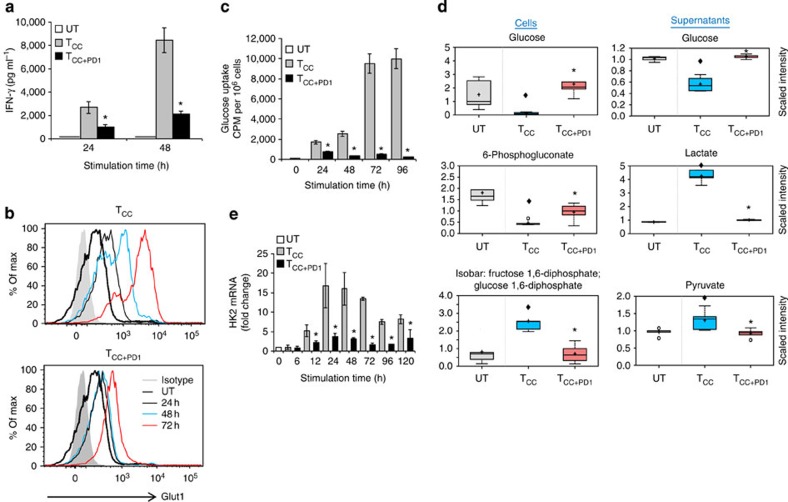
PD-1 inhibits transport and utilization of glucose during T-cell activation. CD4^+^ primary human T cells were either left unstimulated (UT) or were incubated with magnetic beads conjugated with αCD3/αCD28/IgG (T cells co-stimulated (T_CC_)) or magnetic beads conjugated with αCD3/αCD28/PD-L1-Ig fusion protein (T cells co-stimulated+PD-1 (T_CC+PD1_)). (**a**) IFN-γ production was assessed by ELISA (*P<0.05 T_CC+PD1_ versus T_CC_; *n*=3 experiments; Student’s *t*-test). (**b**,**c**) Expression of Glut1 after culture under the indicated conditions and time intervals was examined by flow cytometry, and glucose uptake was examined by 2-{^14^C(U)}-deoxy-D-glucose incorporation. At each time point glucose uptake in T_CC+PD1_-stimulated cells was compared with T_CC_-stimulated cells (*P<0.05, T_CC+PD1_ versus T_CC_; *n*=3 experiments; Student’s *t*-test). (**d**) Analysis of key metabolites involved in glycolysis was performed in cells and culture supernatants as described in Methods. The amounts of the indicated metabolites in unstimulated, T_CC_ and T_CC+PD1_ cells were plotted in whisker boxes. The lower and upper sides of the box indicate the first and third quartile, respectively. The horizontal line inside the box indicates the median value, whereas the lower and upper bars indicate the minimum and maximum of distribution, respectively; (+) mean value; (o) extreme data point. Results of five measurements generated from five independent experiments (*P<0.05, T_CC+PD1_ versus T_CC_, Welch’s *t*-test; ^♦^*P*<0.05, T_CC_ versus UT and T_CC+PD1_ versus UT, analysis of variance). (**e**) HK2 mRNA was analysed by real-time quantitative PCR and relative expression of mRNA of each time point and culture condition over the levels expressed in unstimulated cells (defined as 1) is shown. Analysis was performed over prolonged time of culture (120 h) to investigate whether quantitative difference versus altered kinetics of HK2 expression was induced by PD-1 (*P<0.05, T_CC+PD1_ versus T_CC_, Student’s *t*-test; *n*=3 experiments).

Activation of naïve CD4^+^ T cells is accompanied by effector differentiation, which depends on aerobic glycolysis^13^. To examine the effects of PD-1 on this physiologic mechanism of energy generation on T-cell stimulation, we employed a previously established experimental system^19^ of highly purified human CD4^+^ T cells that were stimulated with magnetic beads conjugated with anti-CD3, anti-CD28 monclonal antibodys (mAbs) and control immunoglobulin-G (IgG; T cells co-stimulated (T_CC_)) or magnetic beads conjugated with anti-CD3, anti-CD28 mAbs and PD-L1-Ig fusion protein (T cells co-stimulated+PD-1 (T_CC+PD1_)), similar to an approach previously used in a mouse cell system^20^. This approach has been designed to mimic the physiologic conditions of primary T-cell activation by antigen-presenting cells (APCs), which leads to engagement of PD-1 and TCR/CD3 complex. Culture of T cells with simultaneous ligation of PD-1 inhibited production of IFN-γ (Fig. 1a), consistent with the known effect of PD-1 signalling on T-cell effector responses.

To uptake glucose from the microenvironment, T cells must upregulate the glucose transporter Glut1 (ref. 8). We determined that Glut1 expression was upregulated in T_CC_, but Glut1 upregulation was less prominent in T_CC+PD1_ cells (Fig. 1b). As a consequence, T_CC_ but not T_CC+PD1_ cells displayed robust glucose uptake (Fig. 1c). Metabolite analysis in T_CC_ cells showed increased glucose utilization for glycolysis, as determined by reduction of intracellular glucose and the pentose phosphate pathway precursor 6-phosphogluconate and increase of the glycolysis intermediate fructose 1,6-bisohosphate. This was accompanied by depletion of glucose and significant elevation in the metabolic products of glycolysis, pyruvate and lactate, in the corresponding culture supernatants (Fig. 1d, Supplementary Fig. 1 and Supplementary Data 1 and 2). PD-1 largely blocked glucose uptake and glycolysis as evidenced by unchanged levels of glucose and diminished products of glycolysis in T_CC+PD1_ cultures (Fig. 1d). This effect of PD-1 was not only due to inhibition of Glut 1 expression and glucose transport but also due to inhibition of hexokinase 2 (HK2) (Fig. 1e), which catalyses the first step of glycolysis by converting glucose to glucose-6-phosphate.

### PD-1 inhibits amino acid transport and metabolism

**Figure 2:**
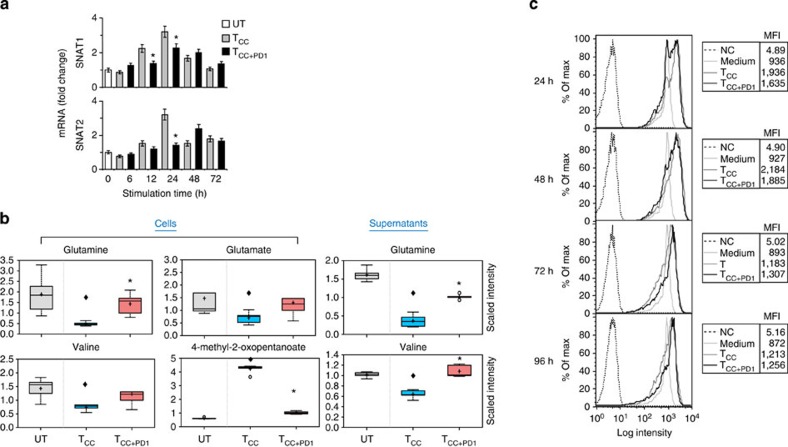
PD-1 inhibits transport and catabolism of glutamine and branched-chain amino acids during T-cell activation. (**a**) SNAT1 and SNAT2 mRNA was analysed by real-time quantitative PCR and relative expression of mRNA of each time point and culture condition over the levels expressed in unstimulated cells (defined as 1) is shown (*P<0.05, T_CC+PD1_ versus T_CC_, Student’s *t*-test; *n*=3 experiments). (**b**) The amounts of glutamine and the glutaminolysis metabolite, glutamate, valine and the metabolic product of valine catabolism, 4-methyl-2-oxopentanoate, were analysed in UT, T_CC_ and T_CC+PD1_ cells and their culture supernatants were plotted in whisker boxes (*P<0.05, T_CC+PD1_ versus T_CC_, Welch’s *t*-test; ^♦^*P*<0.05, T_CC+PD1_ versus UT and T_CC_ versus UT, analysis of variance). Results of five replicate samples generated from five independent experiments. (**c**) Mitochondrial membrane potential (ΔΨ_m_) was assessed after the indicated time intervals of culture by staining with the potentiometric dye TMRE and analysis by flow cytometry; mean fluorescence intensity (MFI) of each sample is shown in the tables. Results are representative of three experiments.

T-cell activation induces changes in amino acid metabolism^21^,^22^. Amino acids serve as building blocks for protein synthesis, but also contribute to many processes critical for dividing cells including nucleotide synthesis, redox control and energy generation. We examined whether PD-1 might alter the ability of T cells to utilize amino acids. Glutamine is a key amino acid involved in numerous processes^23^,^24^. Glutamine catabolism is tightly coupled to various biosynthetic pathways but also generates the anaplerotic substrate a-ketoglutarate, which can be metabolized through the tricarboxylic acid cycle to generate citrate or pyruvate, a process known as ‘glutaminolysis’^24^. The first step of this process involves conversion of glutamine to glutamate, which is subsequently converted to a-ketoglutarate. The main glutamine transporters are members of the SNAT family, SNAT1 and SNAT2, which display a transient increase on T-cell activation^25^. Using real-time quantitative PCR, we determined that SNAT1 and SNAT2 were transiently upregulated in T_CC_ cells but induction was impaired by PD-1 (Fig. 2a). Consistent with the elevated expression of SNAT1 and SNAT2, T_CC_ cells displayed findings of increased glutamine uptake, as determined by decrease of glutamine in the culture supernatants and increased glutaminolysis, as indicated by reduction of intracellular glutamine and glutamate. PD-1 largely blocked glutamine uptake as indicated by glutamine levels in the culture supernatants and inhibited glutaminolysis, as determined by higher intracellular levels of glutamine and glutamate in T_CC+PD1_ compared with T_CC_ cells (Fig. 2b upper row and Supplementary Data 1 and 2).

The branched-chain amino acids (BCAAs) valine, isoleucine and leucine and their metabolites can be incorporated into proteins and/or metabolized to meet energy demands. We examined whether T cells receiving PD-1 signals might metabolize BCAAs. Stimulation of T cells without PD-1 resulted in significant intracellular reduction in BCAAs. Reductions of these BCAAs were also observed in the culture supernatants and coincided with concomitant accumulation of the a-keto acids 4-methyl-2-oxopentanoate, 3-methyl-2-oxovalerate and 3-methyl-2-oxobutyrate, metabolic products of BCAA catabolism (Fig. 2b lower row, Supplementary Fig. 2 and Supplementary Data 1 and 2). Although greater incorporation of BCAAs into proteins to support growth in T_CC_ cells might contribute to the reduction in the levels of these amino acids, the concomitant increase of their degradation products is consistent with increased utilization to meet energy demands. In contrast, T_CC+PD1_ cells displayed neither changes in the levels of BCAA nor changes in the levels of the relevant a-keto acids (Fig. 2b lower row, Supplementary Fig. 2 and Supplementary Data 1 and 2). Thus, on PD-1 ligation T cells are unable to utilize BCAAs for energy demands. In spite of the abrogated glycolysis and amino acid utilization, these T cells remained viable and metabolically active as determined by mitochondrial membrane potential (Fig. 2c).

### PD-1 induces upregulation of CPT1A and FAO

**Figure 3:**
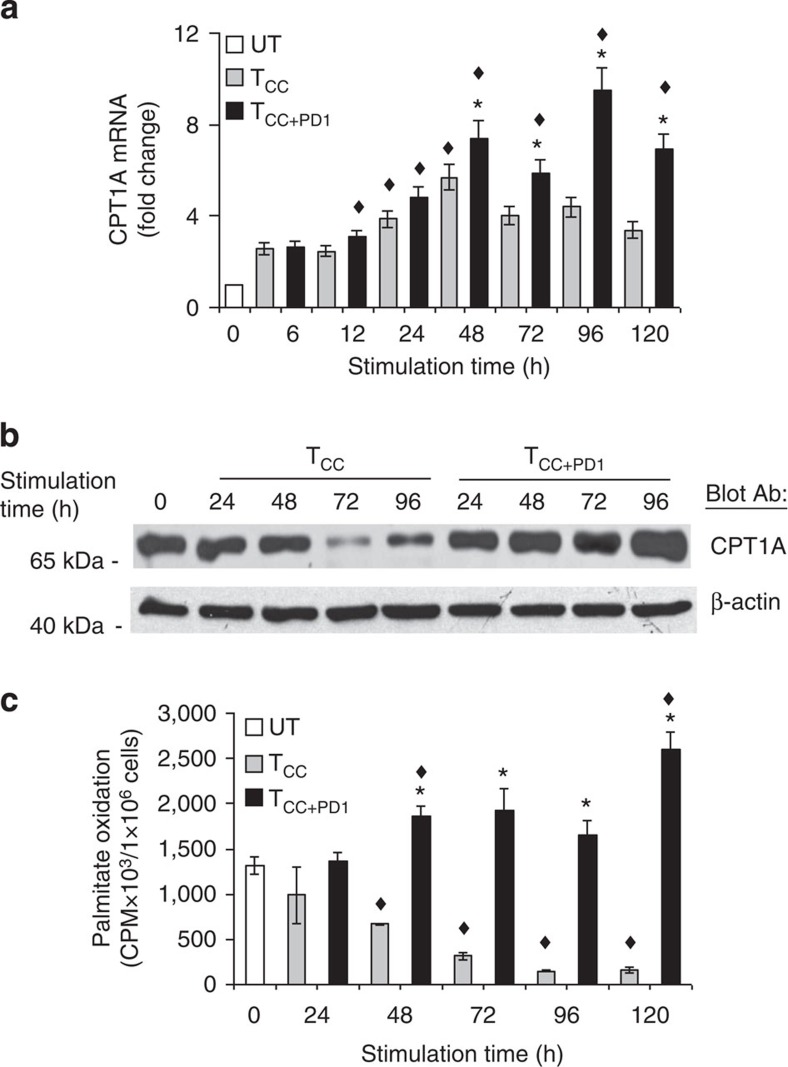
PD-1 induces upregulation of CPT1A and fatty acid β-oxidation. (**a**) CPT1A mRNA was analysed by real-time quantitative PCR and relative expression of mRNA of each time point and culture condition over the levels expressed in unstimulated cells (defined as 1) is shown (*P<0.05, T_CC+PD1_ versus T_CC_, Student’s *t*-test; ^♦^*P*<0.05, T_CC+PD1_ versus UT and T_CC_ versus UT, analysis of variance (ANOVA); *n*=3 experiments). (**b**) CD4^+^ primary human T cells were cultured under the indicated conditions. Cell lysates were prepared at the indicated time points and expression of CPT1A and β-actin were assessed by SDS–PAGE and immunoblot. Results are representative of three experiments. (**c**) Fatty acid β-oxidation rate after culture under the indicated conditions for various time intervals was examined. Values of T_CC_ and T_CC+PD1_ cells were compared with unstimulated (UT) cells (^♦^*P*<0.05, ANOVA; *n*=3) and values of T_CC+PD1_ were compared with T_CC_ cells (*P<0.05, Student’s *t*-test; *n*=3).

A critical pathway targeted by PD-1 is the PI3K/Akt pathway^26^,^27^. Inhibition of the PI3K/Akt pathway by growth factor deprivation in haematopoietic cells activates lipid metabolism through induction of CPT1A^28^, the rate-limiting enzyme of mitochondrial FAO, which plays an important role in the utilization of fatty acids as an energy source. We investigated whether PD-1 might regulate utilization of fatty acids as an alternative energy source. Primary T cells expressed low levels of CPT1A messenger RNA (mRNA), which were rapidly increased by 6 h on stimulation via TCR/CD3 and CD28 in the presence or in the absence of PD-1 ligation. However, the increase of CPT1A mRNA was transient in T_CC_ cells, while in T_CC+PD1_ cells CPT1A mRNA became persistently and progressively elevated compared with unstimulated T cells and T_CC_ cells (Fig. 3a). Furthermore, the abundance of CPT1A protein was diminished on T-cell stimulation via TCR/CD3 and CD28 but was increased by PD-1 (Fig. 3b). We tested whether PD-1 might affect the rate of FAO and determined that unstimulated T cells displayed a baseline rate of FAO, which was decreased by stimulation via TCR/CD3 and CD28 but was upregulated by PD-1 (Fig. 3c).

### PD-1 induces lipolysis and β-oxidation of endogenous fatty acids

**Figure 4:**
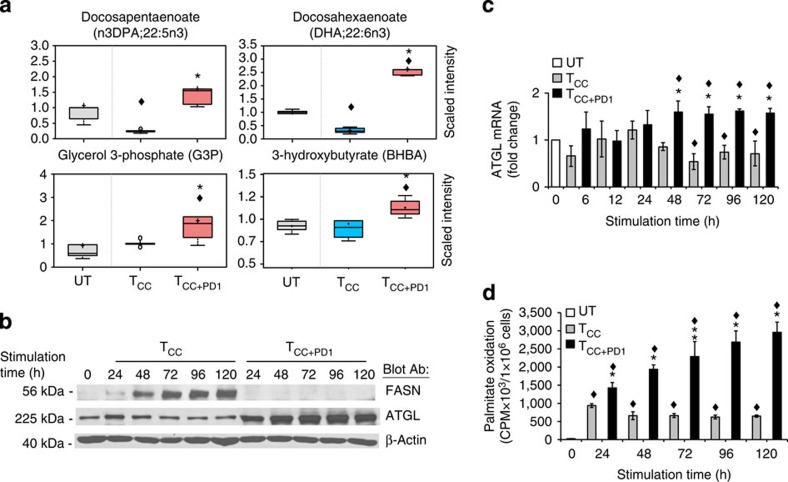
PD-1 induces lipolysis and utilization of endogenous fatty acids for β-oxidation. (**a**) The amounts of n3DPA;22:5n3 and glycerol-3-phosphate (G3P) in the cells and the amounts of DHA;22:6n3 and 3-hyroxybutyrate (BHBA) in the culture supernatants of the indicated culture conditions were analysed and values were plotted in whisker boxes. Values of T_CC_ and T_CC+PD1_ cells were compared with unstimulated (UT) cells (^♦^*P*<0.05, analysis of variance (ANOVA); *n*=5) and values of T_CC+PD1_ were compared with T_CC_ cells (*P<0.05, Welch *t*-test; *n*=5). (**b**) Purified human T cells were cultured under the indicated conditions. Cell lysates were prepared at the indicated time points and expression of FASN, ATGL and β-actin were assessed by SDS–PAGE and immunoblot. Results are representative of three experiments. (**c**) ATGL mRNA was analysed by real-time quantitative PCR and relative expression of mRNA of each time point and culture condition over the levels expressed in unstimulated (UT) cells (defined as 1) is shown. Expression levels of ATGL mRNA in T_CC+PD1_-stimulated cells were compared with TCC-stimulated cells (*P<0.05, Student’s *t*-test) and expression levels in T_CC_ and T_CC+PD1_-stimulated cells were compared with unstimulated (UT) cells (^♦^*P*<0.05, ANOVA). Results are mean±s.e.m. of three experiments. (**d**) T cells were cultured with [9,10-^3^H]palmitate for 48 h, over which time the radiolabelled fatty acid was used to generate cellular lipids. Subsequently, cells were washed to remove unincorporated label and were cultured with beads conjugated with aCD3/CD28/IgG or with beads conjugated with aCD3/CD28/PD-L1-Ig and the amount of β-oxidation of labelled fatty acids generated from endogenous lipid was determined each day. Values of T_CC_ and T_CC+PD1_ cells were compared with unstimulated (UT) cells (^♦^*P*<0.05, ANOVA; *n*=3) and values of T_CC+PD1_ were compared with T_CC_ cells (*P<0.05, Student’s *t*-test; *n*=3).

Metabolite analysis showed that T_CC_ and their culture supernatants had lower quantities of free fatty acids compared with unstimulated cells (Fig. 4a, Supplementary Fig. 3 and Supplementary Data 1 and 2). These changes were likely due to growth of T_CC_ cells, which resulted in greater utilization of free fatty acids for lipid synthesis, incorporation into membrane phospholipids and lipid-based post-translational modification of proteins. Consistent with anabolic lipid metabolism there was also an increase of the fatty acid synthase (FASN) in T_CC_ cells (Fig. 4b). Conversely, T_CC+PD1_ had a small but reproducible increase of several fatty acids compared with unstimulated cells, and significantly higher levels of fatty acids compared with T_CC_ cells (Fig. 4a, Supplementary Fig. 3 and Supplementary Data 1 and 2). We hypothesized that this might be due to the absence of cell growth and utilization of fatty acids for incorporation into membrane phospholipids and lipid-based post-translational modification of proteins because there was no evidence of active lipid synthesis as the induction of FASN was abrogated in T_CC+PD1_ cells (Fig. 4b). However, we also observed that T_CC+PD1_ culture supernatants had increased amounts of the lipolysis marker glycerol (Supplementary Data 2) and T_CC+PD1_ cells had increased amounts of glycerol-3-phosphate, which is generated after phosphorylation of glycerol by glycerol kinase (Fig. 4a), suggesting that PD-1 might induce release of fatty acids from intracellular TG stores. There was also significant elevation of free fatty acids in the culture supernatants (Fig. 4a and Supplementary Data 2).

To examine whether PD-1 might actively promote TG lipolysis, we examined the expression of desnutrin/ATGL, a patatin-domain-containing protein identified as the major TG hydrolase in multiple cell types^29^,^30^,^31^,^32^. Activation of T cells resulted in slight but reproducible decrease of ATGL compared with unstimulated cells (Fig. 4b,c). In contrast, ligation of PD-1 augmented the abundance of ATGL (Fig. 4b,c), suggesting that PD-1 might promote lipolysis and availability of endogenously released fatty acids for FAO.

To investigate this experimentally, we incubated T cells in the presence of ^3^H-labelled palmitate for 48 h, over which time the radiolabelled fatty acid was used to generate cellular lipids. Subsequently, cells were stimulated by the T_CC_ or T_CC+PD1_ protocol. Measurement of the rate of FAO at various time intervals by assessing ^3^H_2_O, the end product of β-oxidation from radiolabelled fatty acids generated from endogenous lipid, revealed a continuous increase of ^3^H_2_O in T cells stimulated with simultaneous PD-1 ligation. In contrast, T cells stimulated without PD-1 displayed a decrease of β-oxidation of endogenous fatty acids, as determined by low levels of ^3^H_2_O that declined steadily (Fig. 4d). Consistent with the increased rate of FAO, PD-1 induced a significant elevation of the ketone body 3-hydroxybutyrate (Fig. 4a and Supplementary Fig. 3), which is produced during FAO. Thus, PD-1 ligation leads to increase of FAO, which coincides with upregulation of ATGL, lipolysis and utilization of endogenous fatty acids for β-oxidation.

### Substantial mitochondrial SRC on PD-1 ligation

**Figure 5:**
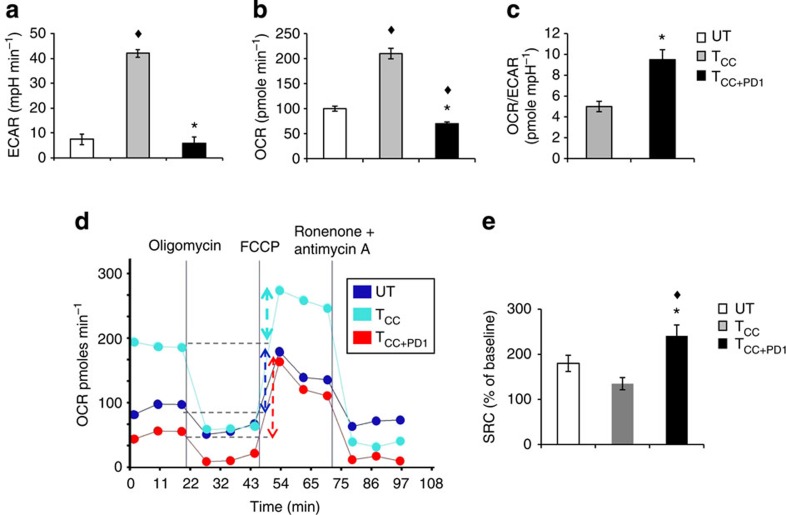
T cells receiving PD-1 signals have substantial mitochondrial spare respiratory capacity. (**a**,**b**) CD4^+^ primary human T cells were cultured under the indicated conditions and 72 h of culture extracellular acidification rate (ECAR) and oxygen consumption rates (OCR) were assessed. (**c**) OCR/ECAR ratio was also measured. (**d**) OCR for each group was measured in real time under basal conditions and after addition of the indicated mitochondrial inhibitors. (**e**) Spare respiratory capacity (SRC) indicated by the difference of maximum OCR over basal OCR (arrows in **d**) calculated as percentage of basal OCR was also determined. Values of T_CC_ and T_CC+PD1_ cells were compared with unstimulated (UT) cells (^♦^*P*<0.05, analysis of variance; *n*=4); values of T_CC+PD1_ cells were compared with T_CC_ cells (*P<0.05, Student’s *t*-test; *n*=4).

To investigate how cellular metabolism of CD4^+^ primary human T cells is regulated during stimulation via TCR/CD3 and CD28 and to determine the role of PD-1, we measured bioenergetic profiles. Extracellular acidification rate (ECAR), an indicator of glycolysis, and oxygen consumption rate (OCR), an indicator of OXPHOS, were increased in T_CC_ in comparison to unstimulated primary T cells (Fig. 5a,b), indicating that activated human CD4^+^ T cells use both glycolysis and OXPHOS, consistent with previous observations in mouse CD4^+^ T cells^13^. T_CC+PD1_ showed lower basal ECAR and OCR (Fig. 5a,b) but had a higher OCR/ECAR ratio compared with T_CC_ (Fig. 5c). These findings indicated that in contrast to proliferating T cells, which preferentially use glycolysis for energy production, T_CC+PD1_ cells are rather metabolically quiescent and preferentially use OXPHOS than glycolysis as indicated by the higher OCR/ECAR ratio. However, after use of FCCP to uncouple ATP synthesis from the electron transport chain, T_CC+PD1_ demonstrated substantial mitochondrial SRC as indicated by the difference between the maximal OCR after FCCP injection and basal OCR, which was higher than the SRC of T_CC_ and of naïve cells (Fig. 5d,e). This was surprising because elevated CPT1A expression and CPT1A-mediated increased SRC are critical regulators of memory T-cell metabolism, survival and function^33^. SRC is the extra mitochondrial capacity available in a cell to produce energy under conditions of increased work or stress and is thought to be important for long-term cell survival^34^. Thus, T cells receiving PD-1 signals have bioenergetic properties of long-lived cells.

### Effects of PD-1 on TCR signaling impact on metabolism

**Figure 6:**
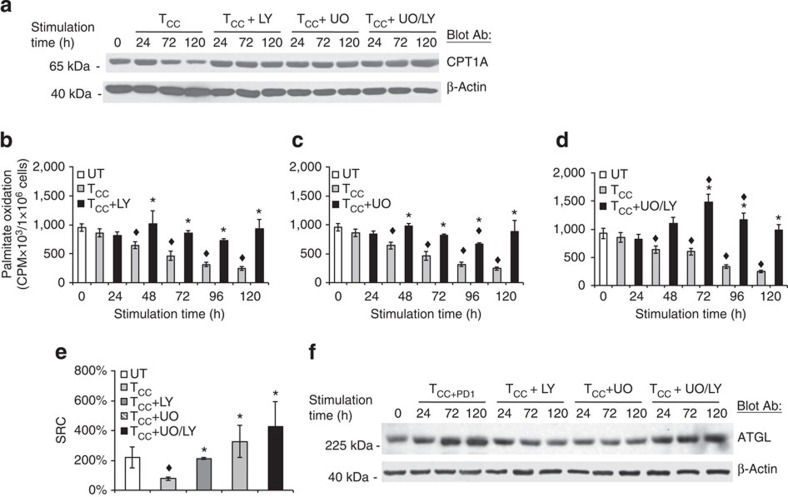
Inhibition of PI3K/Akt and MEK/Erk pathways is involved in the metabolic reprogramming of activated T cells. (**a**) CD4^+^ primary human T cells were cultured with tosyl-activated magnetic beads conjugated with aCD3/aCD28/IgG (T_CC_) in the presence of either LY294002 (LY, 10 μM), UO126 (UO, 10 μM) or their combination. Cell lysates were prepared at the indicated time points and expression of CPT1A and β-actin was assessed by SDS–PAGE and immunoblot. Results are representative of three experiments. (**b**–**d**) In parallel experiments, rate of fatty acid β-oxidation was examined. Values of T_CC_+inhibitor cultures were compared with T_CC_ (*P<0.05, Student’s *t*-test; *n*=3) and values of T_CC_ and T_CC_+inhibitor cultures were compared with unstimulated (UT) (^♦^*P*<0.05, analysis of variance (ANOVA); *n*=3). (**e**) At 72 h of culture under the indicated conditions, spare respiratory capacity (SRC) was determined. Values of T_CC_ and T_CC_+inhibitor cells were compared with unstimulated (UT) cells (^♦^*P*<0.05, ANOVA; *n*=3) and values in T_CC_+inhibitor cells were compared with T_CC_ (*P<0.05, Student’s *t*-test; *n*=3). (**f**) T cells were cultured with tosyl-activated magnetic beads conjugated with aCD3/aCD28/PD-L1-Ig or with tosylatcivated magnetic beads conjugated with aCD3/aCD28/IgG in the presence of either LY294002 (LY, 10 μM), UO126 (UO, 10 μM) or their combination. Cell lysates were prepared at the indicated time points and expression of ATGL and β-actin was assessed by SDS–PAGE and immunoblot.

PI3K/Akt and MEK/Erk pathways have important roles in glucose metabolism^11^,^12^,^35^. Both pathways are targets of PD-1 (refs 19, 27). We examined whether inhibition of these pathways might recapitulate the effects of PD-1 on altering the metabolic reprogramming of activated primary T cells by inhibiting glycolysis and promoting FAO. We used LY294002, a selective inhibitor of PI3K, and UO126, a selective inhibitor of MEK1/2 kinases. Incubation with LY294002 prevented the decrease of CPT1A induced by anti-CD3 and anti-CD28 (Fig. 6a). The decrease of FAO induced on activation was also prevented, resulting in a rate of FAO comparable to unstimulated T cells (Fig. 6b). Incubation with UO126 had similar effects (Fig. 6a,c). Thus inhibition of either PI3K/Akt or MEK/Erk alone, did not fully recapitulate the effects of PD-1, which upregulated both the expression of CPT1A and the rate of FAO. In contrast, simultaneous inhibition of PI3K/Akt and MEK/Erk resulted in increased abundance of CPT1A (Fig. 6a) but also elevated rate of FAO above baseline (Fig. 6d), similar to the effects induced by PD-1. These effects were specific because the use of vehicle control or SB203580, a selective inhibitor of p38 MAPK, did not affect CPT1A expression and FAO (Supplementary Fig. 4a,b). Furthermore, blockade of PD-1 significantly restored Akt and Erk1/2 activation and reduced FAO (Supplementary Fig. 4c,d). Assessment of bioenergetics showed that inhibition of either PI3K/Akt or MEK/Erk suppressed both glycolysis and OXPHOS as determined by diminished ECAR and OCR but promoted preferential OXPHOS utilization as determined by increased OCR/ECAR ratio compared with cells stimulated via TCR/CD3 and CD28 (Supplementary Fig. 5). Concomitantly with the suppression of the glycolytic phenotype, inhibition of PI3K/Akt or MEK/Erk, and more prominently the combined inhibition of PI3K/Akt and MEK/Erk, resulted in increased SRC (Fig. 6e). Incubation with either LY294002 or UO126 alone did not increase the levels of ATGL but the combination of LY294002+UO126 induced ATGL upregulation (Fig. 6f), suggesting that the elevated rate of FAO during simultaneous pharmacologic inhibition of PI3K/Akt and MEK/Erk coincided with increased lipolysis and availability of fatty acids for FAO.

### CTLA-4 inhibits glycolytic metabolism without enhancing FAO

**Figure 7:**
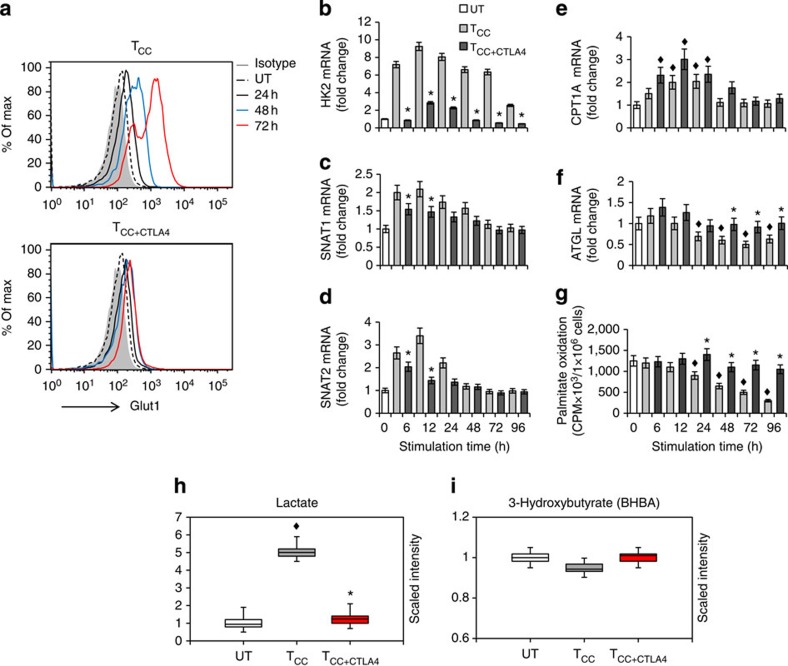
CTLA-4 inhibits glycolytic reprogramming without increasing the rate of fatty acid β-oxidation. CD4^+^ primary human T cells were either left unstimulated (UT) or were incubated with magnetic beads conjugated with αCD3/αCD28/IgG (T cells co-stimulated (T_CC_)) or magnetic beads conjugated with αCD3/αCD28/αCTLA-4 mAbs (T cells co-stimulated+ CTLA-4 (T_CC+CTLA4_)). (**a**) Expression of Glut1 after culture under the indicated conditions and time intervals was examined by flow cytometry. Results are representative of three experiments. (**b**–**f**) mRNA for HK2, SNAT1, SNAT2, CPT1A and ATGL was analysed by real-time quantitative PCR and relative expression of mRNA of each time point and culture condition over the levels expressed in unstimulated cells (defined as 1) is shown. (**g**) Fatty acid β-oxidation rate after culture under the indicated conditions for various time intervals was examined. (**h**,**i**) Analysis of lactate (**h**) and 3-hyroxybutyrate (**i**), end metabolites of glycolysis and fatty acid β-oxidation, respectively, was performed in culture supernatants. (For the studies shown in all panels: *P<0.05, T_CC+CTLA4_ versus T_CC_, Student’s *t*-test; ^♦^*P*<0.05, T_CC_ versus UT and T_CC+CTLA4_ versus UT, analysis of variance; *n*=3 experiments).

CTLA-4 is another critical T-cell inhibitor of the B7/CD28 superfamily. We examined whether engagement of CTLA-4 would have similar implications on metabolic reprogramming of T cells, as PD-1. We used a CTLA-4 agonist antibody that mediates inhibitory effects on T cells when conjugated on tosyl-activated magnetic beads together with anti-CD3 antibodies with or without anti-CD28 (ref. 36). First, we examined expression of Glut1, which is induced on T-cell stimulation and is a key determinant of glycolytic metabolism. CTLA-4 engagement inhibited Glut1 expression (Fig. 7a), and this was even more pronounced than the inhibition of Glut1 induced by PD-1 (Fig. 1b). Induction of HK2, the rate-limiting enzyme of glycolysis, was also diminished (Fig. 7b). As a consequence, T cells receiving CTLA-4 signals did not activate glycolytic metabolism, as determined by lack of lactate elevation (Fig. 7h). CTLA-4 also suppressed the expression of the glutamine transporters SNAT1 and SNAT2 (Fig. 7c,d), indicating the uptake and, therefore, metabolism of glutamine were not activated. Although these effects were comparable with those induced by PD-1, CTLA-4 mediated a distinct effect on the regulators of lipid metabolism CPT1A and ATGL. Compared with baseline, CPT1A and ATGL mRNA displayed a small, transient increase, (Fig. 7e,f) but these elevated mRNAs did not reach the magnitude or the persistence induced by PD-1 ligation (compare Fig. 7e,f with Figs 3a and 4c). Furthermore, ligation of CTLA-4 did not result in detectable changes in the rate of FAO as compared with unstimulated cells (Fig. 7g) and there was no detectable increase of 3-hydroxybutyrate in the culture supernatants (Fig. 7i). These results indicate that, in contrast to PD-1, engagement of CTLA-4 does not imprint a pattern of altered metabolic reprogramming but rather preserves the metabolic profile of unstimulated T cells.

### PD-1 switches the glycolytic programme of pre-activated T cells

PD-1 is considered an activation-induced surface molecule because it is not easily detected in resting T cells but is maximally expressed by 48 h of activation. However, effects of PD-1 ligation can be observed by adding PD-L1-Ig fusion protein as early as a few hours after activation or even at the initiation of the culture, suggesting that functional levels of PD-1 might be expressed very early after T-cell activation. To examine whether PD-1-mediated metabolic reprogramming might be related to the distinct levels of PD-1 expression at different time points of T-cell activation, first we performed a detailed analysis of PD-1 kinetics of surface protein expression at 2, 4, 8 and 24 h post stimulation and subsequently we engaged PD-1 and examined its effects on T-cell metabolism. Consistent with a previous similarly detailed analysis^37^, we detected only minimal PD-1 surface expression until 8 h of culture, which slightly increased at 24 h, peaked at 48 h and subsequently declined (Supplementary Fig. 6). Because comparable PD-1 expression was detected during 2–8 h of culture, we pre-activated T cells for 4 or 24 h and subsequently re-cultured them in the presence of PD-L1-Ig and examined effects on metabolism.

**Figure 8:**
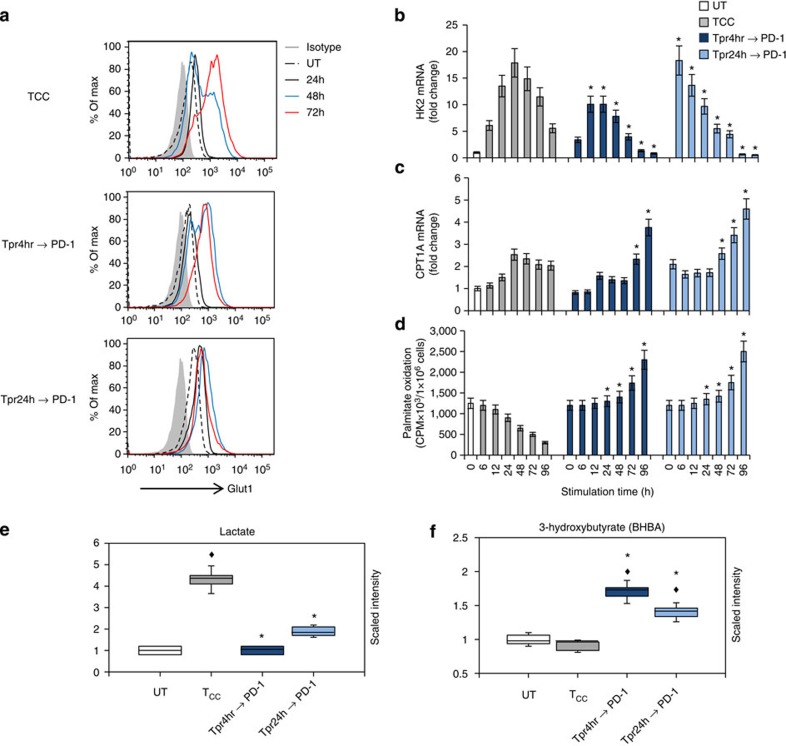
PD-1 alters the metabolic programme of activated T cells from glycolysis to FAO. CD4^+^ human T cells were pre-activated with anti-CD3-and-anti-CD28 mAbs for 4 h or for 24 h and subsequently were collected, were left to rest for 3 h and re-cultured with tosyl-activated magnetic beads conjugated with αCD3/αCD28/PD-L1-Ig (Tpr4hr→PD-1 and Tpr24hr→PD-1). In the same experiment CD4^+^ T cells without pre-activation were stimulated with tosyl-activated magnetic beads conjugated with αCD3/αCD28/IgG (T_CC_) and used as positive control for optimal stimulation without PD-1. (**a**) Expression of Glut1 after culture under the indicated conditions and time intervals was examined by flow cytometry. (**b**,**c**) mRNA for HK2 and CPT1A was analysed by real-time quantitative PCR and relative expression of mRNA of each time point and culture condition over the levels expressed in unstimulated cells (defined as 1) is shown. (**d**) Fatty acid β-oxidation rate after culture under the indicated conditions for various time intervals was examined. (**e**,**f**) Analysis of lactate (**e**) and 3-hydroxybutyrate (**f**), end metabolites of glycolysis and fatty acid β-oxidation, respectively, was performed in culture supernatants (for the studies shown in all panels: *P<0.05, Tpr4hr→PD-1 versus T_CC_ or Tpr4hr→PD-1 versus T_CC_, Student’s *t*-test; ^♦^*P*<0.05, T_CC_ versus UT, Tpr4hr→PD-1 versus UT and Tpr4hr→PD-1 versus UT, analysis of variance; *n*=3 experiments).

After 4 h of pre-activation, there was no detectable increase of Glut 1 expression but PD-1 ligation in these pre-activated T cells diminished subsequent Glut1 upregulation, as compared with control non pre-activated cells stimulated by anti-CD3 and anti-CD28 (Fig. 8a, middle and top panels). Strikingly, a robust suppression effect on Glut1 was observed in T cells that had been pre-activated for 24 h, in which Glut1 expression was already induced (Fig. 8a, bottom panel). Although HK2 mRNA was elevated in 4 h pre-activated T cells, and even more so in 24 h pre-activated T cells PD-1 ligation diminished HK2 expression at all time points of re-culture (Fig. 8b). As a consequence, pre-activated T cells turned off their glycolytic metabolism, resulting in reduced levels of lactate in the culture supernatants (Fig. 8e). In contrast, under these conditions, pre-activated T cells elevated CPT1A expression (Fig. 8c) and displayed increased rate of FAO and elevated production of 3-hyroxybutyrate (Fig. 8d,f). Furthermore, PD-1 ligation in pre-activated T cells reversed the decrease of ATGL and restored its expression to levels higher than in T cells stimulated with anti-CD3 and anti-CD28 (Supplementary Fig. 7c). PD-1 also suppressed the expression of the glutamine transporters SNAT1 and SNAT2 in pre-activated T cells (Supplementary Fig. 7a,b). These results indicate that PD-1 does not simply prevent initiation of glycolytic metabolism but imprints a pattern of metabolic reprogramming that can be induced even after an initial pre-activation phase in the absence of PD-1 ligation.

## Discussion

In the present study, we examined the effects of PD-1 on the metabolic programme and bioenergetics of activated CD4^+^ T cells. Our findings revealed that T cells receiving PD-1 signals were unable to uptake and utilize glucose, branched-chain amino acids and glutamine but were metabolically active and had an increased rate of FAO. This event was associated with increased expression of the major TG hydrolase ATGL and the key enzyme of FAO CPT1A. These concomitant biochemical and molecular events resulted in increased availability and utilization of free fatty acids for energy generation through FAO. Furthermore, T cells receiving PD-1 signals had increased SRC, the extra capacity available in cells to produce energy under increased stress or work, which is associated with cell survival. The metabolic and bioenergetic properties of PD-1-stimulated T cells display an unexpected and surprising similarity to those of memory T cells, which sustain their survival due to catabolic metabolism of FAO^33^.

In contrast to PD-1, CTLA-4 did not induce a distinct metabolic programme but preserved the metabolic profile of non-stimulated cells. The distinct properties of CTLA-4 and PD-1 in regulating metabolic reprogramming of T cells might be related to the differential ability of these receptors to oppose TCR signals. For example, CTLA-4 mediates a potent inhibition of T-cell activation and its recruitment at the immunological synapse is regulated by the strength of the TCR signal^38^. In contrast, PD-1 can inhibit weak but not strong TCR signals^39^ and, as a consequence certain but not all TCR-mediated biochemical events are blocked^19^. The selective effects of PD-1 on TCR proximal signalling might have an active role in the regulation of a distinct metabolic reprogramming, which also leads to a distinct functional fate.

Our studies revealed that increase of fatty acids, the substrate of FAO, as well as generation of the ketone body 3-hyroxybutyrate, a product of FAO, were observed during T-cell stimulation with simultaneous ligation of PD-1. Fatty acids are important metabolic intermediates, because they can either be used for lipid synthesis and protein modification or can be degraded through mitochondrial β-oxidation for energy generation. Our biochemical studies and our metabolite analysis revealed that in the presence of PD-1 ligation, T cells had elevated levels of the lipolysis marker glycerol and increased abundance of ATGL, and displayed increased utilization of endogenous lipids for β-oxidation. In contrast, ATGL was rather reduced in T cells stimulated without PD-1, and these cells had diminished utilization of endogenous lipids for β-oxidation.

Recent studies determined that T effector cells have the capacity to acquire exogenous fatty acids, whereas T-memory cells do not acquire fatty acids but, instead, synthesize fatty acids by using glucose-derived carbon. Possibly these fatty acids are then converted into TGs and cholesterol esters, which undergo hydrolysis mediated by lysosomal acid lipase (LAL), thereby generating free fatty acids for FAO^40^. In contrast to the findings in memory cells, we did not detect increase of LAL in PD-1-stimulated cells. Instead, LAL was upregulated in T cells undergoing optimal stimulation and this was inhibited by PD-1 (K.B. and V.A.B., unpublished observations). These findings might be consistent with a specific role of LAL in regulating fatty acid availability in memory T cells, which have been previously activated during antigen encounter.

Although previously thought to play a role only in adipocytes where it releases fatty acids from triacylglycerols stored in lipid droplets, it is today known that ATGL is widely expressed in various mammalian cell types^41^,^42^. Lipid droplets are present essentially in all cell types, including those for which energy storage does not seem to be their main purpose. Lipid droplets, particularly in nonadipose tissues, undergo dynamic and rapid changes of formation and degradation^43^. ATGL-mediated lipolysis also has a key role in mitochondrial homeostasis by generating PPAR ligands, which induce PPAR-mediated expression of oxidative genes^32^,^44^. Cellular fatty acids obtained from extracellular sources or generated by *de novo* synthesis, require esterification to TGs and subsequent lipolysis by ATGL before initiating signal transduction to the nucleus^43^. Because naïve T cells rely on FAO for energy generation, it is possible that ATGL has a key role in regulating fatty acid availability for FAO and expression of oxidative genes under conditions of limited fatty acid uptake and lipid synthesis. In contrast, LAL is upregulated in activated T cells, which dramatically increase their ability to uptake fatty acids and to synthesize lipids, which are subsequently stored as cholesterol esters and TGs in the lysosomes. Elevated LAL levels might persist or further increase after the glycolytic metabolism of the effector phase is turned off and the surviving effectors develop into memory cells, which relay on FAO but obtain biochemical and bioenergetic properties distinct from naïve T cells. Further work will be required to identify the functions of these and possibly other lipases in the distinct phases of T-cell differentiation.

An intriguing observation of our metabolite analysis was the significant elevation of polyunsaturated fatty acids when T cells are stimulated with PD-1 ligation. Polyunsaturated fatty acids such as omega-3 fatty acids are recognized as modulators of immunity. Although the molecular mechanisms are incompletely understood, polyunsaturated fatty acids seem to suppress antigen presentation, T-cell activation and cytokine production^45^,^46^. These fatty acids can also alter composition and organization of membrane rafts with a consequent impact on the activity of raft-associated signalling proteins and downstream events^47^. It will be important to investigate in future work whether such polyunsaturated fatty acids are involved in PD-1-mediated cell intrinsic or cell extrinsic functional effects.

Persistent ligation of PD-1 enforces T-cell exhaustion, a state of T-cell dysfunction that arises during chronic infections and cancer^48^,^49^,^50^. T-cell exhaustion is an active process and can lead to measurable consequences on T-cell function. For example, distinct subsets of exhausted T cells exist with different potentials for recovering function after blockade of the PD-1 pathway. Exhausted T cells with intermediate expression of PD-1 (PD-1^int^) cells can be reinvigorated by blockade of the PD-1 pathway, whereas those with high expression of PD-1 (PD-1^high^ cells) cannot^51^. It is possible that these distinct subsets of exhausted T cells may also have different bioenergetic properties and differential capacity for mitochondrial biogenesis, which may correlate with SRC, CPT1A and ATGL expression. It is tempting to speculate that the degree of exhaustion and the ability to reinvigorate exhausted T cells might depend on the reserve of lipids, which appear to be the only source of energy generation by FAO in T cells receiving PD-1 signals. Identification of such distinct bioenergetic profiles in exhausted T-cell subsets might provide new tools to determine the level of T-cell exhaustion. It may also provide novel targets to reverse exhaustion in addition to PD-1 blockade. Future studies will address these issues in the context of PD-1-mediated T-cell exhaustion *in vivo*.

Based on our findings it is also possible that PD-1-mediated inhibitory effects on T-cell function might be related to a more oxidative environment, potentially due to increased β-oxidation. Our metabolite analysis showed that although depletion of the cellular antioxidant glutathione (GSH) was observed in T cells stimulated in the presence or in the absence of PD-1 ligation, consistent with the expected increase of reactive oxygen species on activation^52^,^53^, PD-1 ligation resulted in significantly more pronounced decrease in the levels of reduced GSH (Supplementary Data 1). However, these cells displayed higher levels of cysteine-GSH disulfide and ophthalmate, a GSH-like product synthesized by the same enzymes. These changes indicate a higher attempt to increase GSH synthesis, which, together with the more pronounced decrease in the levels of reduced GSH, are suggestive of a more oxidative environment in T cells receiving PD-1 signals. A key mediator of oxidative detoxification is the PPARγ coactivator-1a (PGC-1a)^54^,^55^. Induction of PGC-1a during caloric restriction mediates adaptations to provide a permissive setting for increased OXPHOS. Such changes include the expression of uncoupling proteins, which have been associated with decreased oxidation-induced damage. Importantly, mammalian target of rapamycin, a target of PD-1 downstream of PI3K/Akt inhibition, has a direct effect on PGC-1a expression^56^. Thus, PD-1 might impair oxidative detoxification. Future studies will determine how PGC-1a is regulated on T-cell activation and whether PD-1 might suppress the expression and function of PGC-1a.

In conclusion our present studies show that PD-1 induces metabolic reprogramming of TCR-stimulated T cells from glycolysis to lipolysis and utilization of endogenous free fatty acids for β-oxidation. This programme provides a means for survival of T cells receiving PD-1 signals during their reduced capacity for uptake and utilization of other nutrient classes. These findings open new directions for the mechanistic understanding of the impaired T-cell effector function mediated by PD-1 and identify novel targets that can be exploited therapeutically to overcome the implications of PD-1 signalling.

## Methods

### *In vitro* T-cell cultures

Leucocytes were obtained from normal healthy blood donors. Primary CD4^+^ T cells were isolated by negative selection using the CD4^+^ T cell Isolation Kit II from Miltenyi Biotec (Auburn, CA) and cultured at 1:1 ratio with tosyl-activated magnetic beads (1.5 × 10^5^ beads per well) conjugated either with anti-CD3, anti-CD28 mAbs and IgG2a or with anti-CD3, anti-CD28 and PD-L1-IgG2a (ref. 26). For the preparation of Dynabeads M-450 (Invitrogen) tosyl-activated magnetic beads the following mAbs were used: anti-CD3 (UCHT1, R&D Systems, Inc.), anti-CD28 (CD28.2, Biolegend) and either PD-L1-IgG2a (ref. 39) or control IgG2a (Jackson Immunoresearch Laboratories, West Grove, PA). About 2 × 10^8^ magnetic beads were coated with anti-CD3 (8%), anti-CD28 (10%) and either PD-L1-Ig fusion protein or control IgG comprised the remaining 82% of the total protein. All incubations were performed in 0.1 M sodium phosphate buffer for 18 h at 37 °C with constant rotation. The beads were then washed three times and stored at 4 °C. DNA synthesis was assessed by [^3^H]-thymidine incorporation for the last 16 to 18 h of a 72-h culture. In pre-activation and subsequent PD-1 ligation experiments, CD4^+^ T cells were cultured with anti-CD3-and-anti-CD28 mAbs for the indicated time intervals and subsequently were collected, were left to rest in culture media for 3 h and re-cultured with tosyl-activated beads (1.5 × 10^5^ beads per well) conjugated with anti-CD3, anti-CD28 mAbs and PD-L1-IgG2a. In the same experiment, CD4^+^ T cells without pre-activation were cultured with tosyl-activated magnetic beads (1.5 × 10^5^ beads per well) conjugated with anti-CD3, anti-CD28 mAbs and IgG2a and were used as a positive control. When indicated, the blocking anti-PD1 antibody J105 (eBioscience) was used. For studies of CTLA-4-mediated effects on T-cell metabolic reprogramming, the CTLA-4 antibody 14D3 (eBioscience) was used to coat on tosyl-activated magnetic beads instead of PD-L-1-Ig. Where indicated, cells were cultured in the presence of the PI3K inhibitor LY294002 (10 μM), the MEKK inhibitor UO126 (10 μM), the p38 MAPK inhibitor SB203580 (10 μM) or the combination of LY294002 and UO126 (all inhibitors from Sigma-Aldrich, St Louis, MO), using a concentration previously determined to induce target-specific inhibition without affecting cell viability^57^.

### Immunoblotting

Cell lysates were prepared as previously mentioned^19^ and equal amounts of protein of each sample were analysed by SDS–PAGE. The antibodies for Glut1 (sc-1603), ATGL (sc-365278) and β-actin (sc-1615) were from Santa Cruz Biotechnology, Santa Cruz, CA. The anti-FASN (#3189) was from Cell Signaling Technologies, Danvers, MA, and the anti-CPT1A (#TA303144) antibody was from OriGene Technologies Inc., Rockville, MD.

### Assessment of Glut1 expression by flow cytometry

T cells were stimulated for the indicated time intervals with bead-linked Abs and fixed overnight at 4 °C with Fixation Buffer (eBioscience). Fixed cells were permeabilized with permeabilization buffer (eBioscience) and stained with FITC-conjugated anti-Glut1 Ab (R&D Systems, Inc.). Mouse IgG2b was used as an isotype control. Glut1 staining was measured by flow cytometry on BD LSRII, and data were analysed with FlowJo software (Tree Star Inc).

### Glucose uptake assay

T cells were cultured at the indicated conditions and then 1 × 10^6^ cells were starved in PBS at room temperature for 30 min followed by incubation at 37 °C for 5 min in PBS containing 5 μM 2{^14^C(U)}-deoxy-D-glucose (Perkin Elmer, Boston, MA)^58^. Cells were then harvested on filtermats and counted for ^14^C-glucose content.

### β-oxidation assay

T cells were cultured at the indicated conditions at 1 × 10^6^ cells per ml. Oxidation of fatty acids was measured according to established method^28^. Briefly, cells were supplemented with [9,10-^3^H]palmitate (Perkin Elmer, Boston, MA) complexed to BSA by vortexing for 1 min a mixture of the palmitate and a 10% fatty acid-free BSA (Sigma, St Louis, MO #A8806) solution at a 1:2 volume ratio. A total of 3.3 μl of [9,10-^3^H]palmitate (5 μCi μl^−1^ stock) and 6.7 μl of BSA were used per 1 ml of cell culture medium and cells were cultured in 24-well plates with or without etomoxir. After 24 h, 0.4 ml supernatant was applied to ion-exchange columns (Dowex 1 × 8–200, Sigma, #217425), and ^3^H_2_O was recovered by eluting with 1 ml of H_2_O. A 0.4-ml aliquot was then mixed with 1 ml scintillation fluid (Ultima Gold, #6013321, Perkin Elmer) and counted. Cell number was counted after the collection of each supernatant and the results were expressed as CPM × 10^3^/1 × 10^6^ cells. To measure utilization of endogenous lipids as a substrate of FAO, we incubated primary human T cells (40 × 10^6^) in the presence of phytohaemaglutinin (10 μg ml^−1^), phorbol myristate (1 ng ml^−1^) and tritium-labelled palmitate for 48 h. This mixture was prepared by adding 20 μCi of [9,10-^3^H]palmitate (5 μCi μl^−1^ stock) to 1 ml of 10 μM unlabelled palmitate stock in a solution of 10% essentially fatty acid-free BSA into 20 ml culture medium for 48 h, over which time the radiolabelled fatty acid was used to generate cellular lipids. Subsequently, cells were washed to remove unincorporated label and were stimulated with beads conjugated with anti-CD3, anti-CD28 mAbs and IgG or with beads conjugated with anti-CD3, anti-CD28 mAbs and PD-L1-Ig fusion protein, with or without etomoxir. Culture supernatants were collected at various time intervals and 0.4 ml supernatant was applied to ion-exchange columns and determination of radioactivity was performed as described above.

### Metabolism assays

OCR and ECAR were measured according to previously established protocol^33^ using XF base media (#102353-100) supplemented with 25 mM glucose, 2 mM L-glutamine and 1 mM sodium pyruvate under basal conditions, and in response to 1 mM oligomycin, 1.5 mM fluorocarbonyl cyanide phenylhydrazone and 100 nM rotenone+1 mM antimycin A using the XF Cell Mito Stress Test Kit on a XF-24 Extracellular Flux Analyzer (Seahorse Bioscience, North Billerica, MA).

### Metabolite analysis

T cells were grown under the indicated culture conditions at 4 × 10^6^ cells per 6 ml per well in six-well culture plates. After 96 h of culture, 30 × 10^6^ cells and 0.5 ml culture supernatants were harvested separately, flash-frozen in liquid nitrogen and stored at −80 °C. A total of five replicate samples for each condition generated from five independent experiments were analysed on Gas chromatography–mass spectrometry (GC/MS) and liquid chromatography–tandem mass spectrometry (LC/MS/MS) platforms^59^,^60^ by Metabolon Inc. (Durham, NC), as described below.

*Sample accessioning*. Each sample received was accessioned into the Metabolon Laboratory Information Management System (LIMS) and was assigned by the LIMS a unique identifier, which was associated with the original source identifier only. This identifier was used to track all sample handling, tasks, results and so on. The samples (and all derived aliquots) were bar coded and tracked by the LIMS system. All portions of any sample were automatically assigned their own unique identifiers by the LIMS when a new task was created; the relationship of these samples was also tracked. All samples were maintained at −80 °C until processed.

*Sample preparation*. The sample preparation process was carried out using the automated MicroLab STAR system from Hamilton Company. Recovery standards were added prior to the first step in the extraction process for quality control (QC) purposes. Sample preparation was conducted using a proprietary series of organic and aqueous extractions to remove the protein fraction, while allowing maximum recovery of small molecules. The resulting extract was divided into two fractions; one for analysis by LC and one for analysis by GC. Samples were placed briefly on a TurboVap (Zymark) to remove the organic solvent. Each sample was then frozen and dried under vacuum. Samples were then prepared for the appropriate instrument, either using LC/MS or GC/MS.

*Ultra-performance liquid chromatography–tandem mass spectrometry*. The LC/MS portion of the platform was based on a Waters ACQUITY UPLC and a Thermo-Finnigan LTQ mass spectrometer, which consisted of an electrospray ionization source and linear ion-trap mass analyser. The sample extract was split into two aliquots, dried, then reconstituted in acidic or basic LC-compatible solvents, each of which contained ≥11 injection standards at fixed concentrations. One aliquot was analysed using acidic positive ion optimized conditions and the other using basic negative ion optimized conditions in two independent injections using separate dedicated columns. Extracts reconstituted in acidic conditions were gradient eluted using water and methanol both containing 0.1% formic acid, while the basic extracts, which also used water/methanol, contained 6.5 mM ammonium bicarbonate. The MS analysis alternated between MS and data-dependent MS^2^ scans using dynamic exclusion.

*Gas chromatography/mass spectrometry*. The samples destined for GC/MS analysis were re-dried under vacuum desiccation for a minimum of 24 h prior to being derivatized under dried nitrogen using bistrimethyl-silyl-triflouroacetamide. The GC column was 5% phenyl and the temperature ramp is from 40° to 300 °C in a 16-min period. Samples were analysed on a Thermo-Finnigan Trace DSQ fast-scanning single-quadrupole mass spectrometer using electron impact ionization. The instrument was tuned and calibrated for mass resolution and mass accuracy on a daily basis. The information output from the raw data files was automatically extracted as discussed below.

*Data extraction, quality assurance/QC and compound identification*. The data extraction of the raw mass spec data files yielded information that could be loaded into a relational database. Once in the database, the information was examined and appropriate QC limits were imposed. Peaks were identified using Metabolon’s proprietary peak integration software. Compounds were identified by comparison to library entries of purified standards or recurrent unknown entities. Identification of known chemical entities was based on comparison to metabolomic library entries of purified standards.

*Statistical calculation*. For pair-wise comparisons Welch’s *t*-test was used. Analysis of variance was used for comparison of more than two conditions. Selected metabolites were plotted in whisker boxes.

### Real-time qPCR

Total RNA extraction was prepared with the RNeasy Mini Kit from Qiagen, Valencia, CA, according to the manufacturer’s instructions and 50 ng of RNA was subjected to qPCR analysis as described previously^19^ for the target genes HK2, CPT1A, SNAT1, SNAT2 and ATGL. All FAM-conjugated gene-specific primers as well as the TaqMan One-Step RT-PCR Master Mix reagents and the VIC-TAMRA-conjugated 18S RNA housekeeping gene control primers were from Applied Biosystems/Roche, Branchburg, NJ. The reaction was performed in a AB 7000 quantitative PCR machine from Applied Biosystems.

### Assessment of mitochondrial membrane potential (ΔΨ_m_)

Cells were harvested, stained in culture medium with TMRE (Sigma-Aldrich) to a final concentration of 100 nM, and incubated for 30 min at 37 °C with 5% CO_2_. CCCP (Sigma-Aldrich) was added to collapse ΔΨ_m_ and therefore to validate the assay and serve as a control for background levels of fluorescence. Cells were analysed for TMRE intensity by flow cytometry.

### Cytokine production

Culture supernatants were harvested at the indicated time points and the concentration of IFN-γ was assessed by ELISA using the high sensitivity assay kit (eBioscience, Inc. San Diego, CA) according to the manufacturer’s instructions.

### Statistical analysis

*In vitro* assays were compared with the unpaired Student’s *t*-test or by analysis of variance for more than two conditions. A *P* value of <0.05 was considered statistically significant.

## Author contributions

N.P., K.B., P.C., D.S., B.L., L.L. and V.A.B. performed experiments in various aspects of the project; V.P. assisted with qPCR experiments; G.J.F. provided PD-L1-IgG2 fusion protein; L.N.B. and E.D.K. performed metabolite analysis; P.S. assisted with the bioenergetics studies; V.A.B. guided the studies; all authors contributed to the preparation of the manuscript.

## Additional information

**How to cite this article:** Patsoukis, N. *et al*. PD-1 alters T-cell metabolic reprogramming by inhibiting glycolysis and promoting lipolysis and fatty acid oxidation. *Nat. Commun.* 6:6692 doi: 10.1038/ncomms7692 (2015).

## Supplementary Material

Supplementary Figures Supplementary Figures 1-7

Supplementary Data 1 T cells were cultured either in medium (UT) or with magnetic beads conjugated with anti-CD3, anti-CD28 mAbs and control IgG (T cells costimulated; TCC) or with magnetic beads conjugated with anti-CD3, anti-CD28 mAbs and PD-L1-Ig fusion protein (T cells costimulated+PD-1; TCC+PD1). After 96 hours, cells (30 x 106/sample) were harvested and metabolites were analyzed. Five replicate samples for each condition were generated from five independent experiments.

Supplementary Data 2 T cells were cultured either in medium (UT) or with magnetic beads conjugated with anti-CD3, anti-CD28 mAbs and control IgG (T cells costimulated; TCC) or with magnetic beads conjugated with anti-CD3, anti-CD28 mAbs and PD-L1-Ig fusion protein (T cells costimulated+PD-1; TCC+PD1). After 96 hours, culture supernatants (500 ul/sample) were harvested and metabolites were analyzed. Five replicate samples for each condition were generated from five independent experiments.
