# Supplementary material for: PD-1 alters T-cell metabolic reprogramming by inhibiting glycolysis and promoting lipolysis and fatty acid oxidation
Source: Nat Commun. 2015 Mar 26;6:6692. doi: 10.1038/ncomms7692 (PMC4389235; doi:10.1038/ncomms7692)
Supplement: Supplementary Figures — 1-7 [file ncomms7692-s1.pdf]

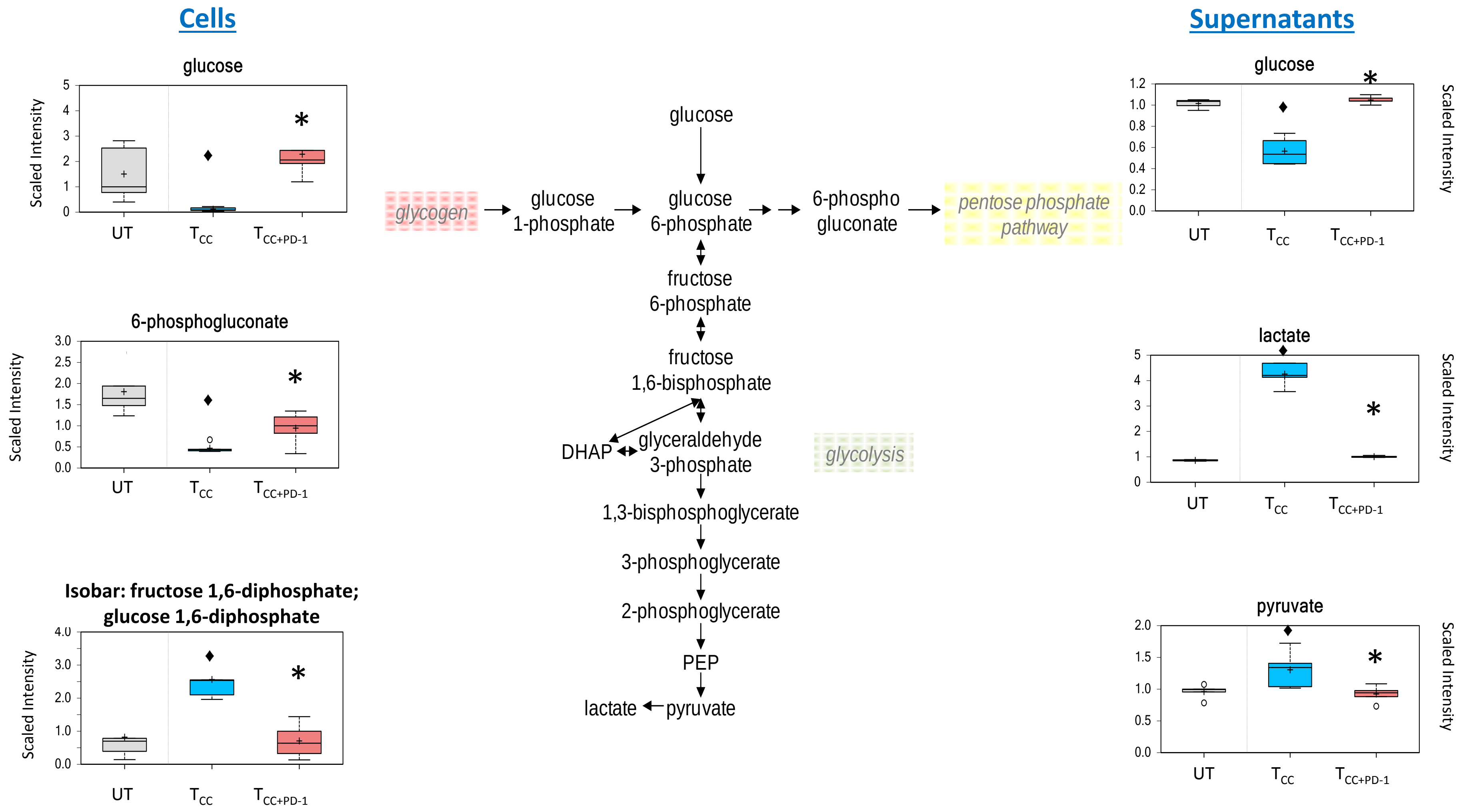

Supplementary figure 1. PD-1 inhibits glycolytic metabolism during T cell activation. Analysis of key metabolites involved in glycolysis was performed in cells and culture supernatants as described in Materials and Methods. The amounts of the indicated metabolites in unstimulated, T_{CC} and T_{CC+PD1} cells were plotted in whisker boxes. The glycolytic pathway is shown in parallel.

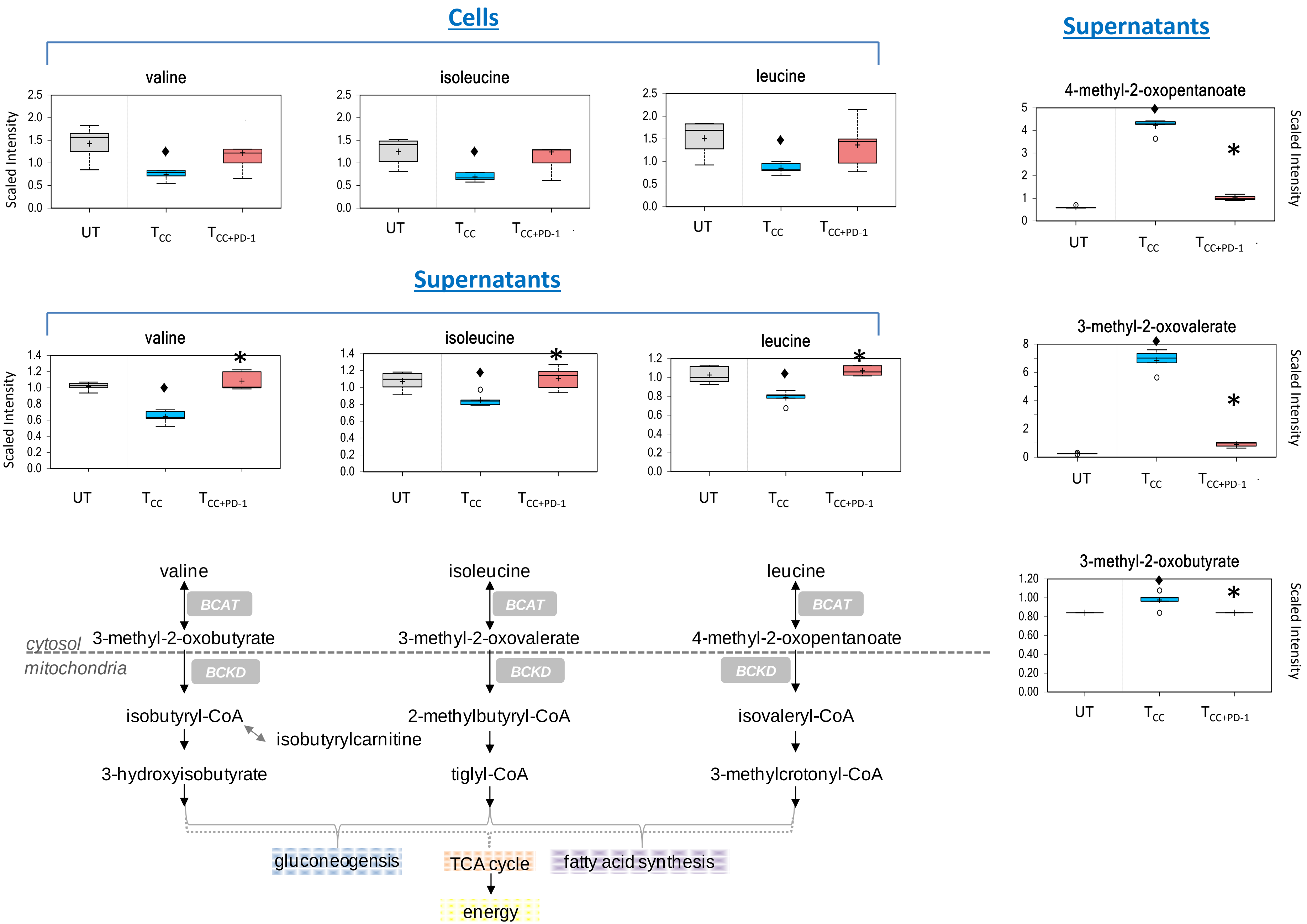

Supplementary figure 2. PD-1 inhibits utilization of branched-chain amino acids during T cell activation. The quantities of valine, isoleucine and leucine and the relevant keto-acids generated upon metabolic utilization of the branched-chain amino acids were analyzed in UT, T_{CC} and $T_{CC+PD-1}$ cells and their culture supernatants and were plotted in whisker boxes. The integration steps of these keto-acids in metabolic pathways are shown in parallel.

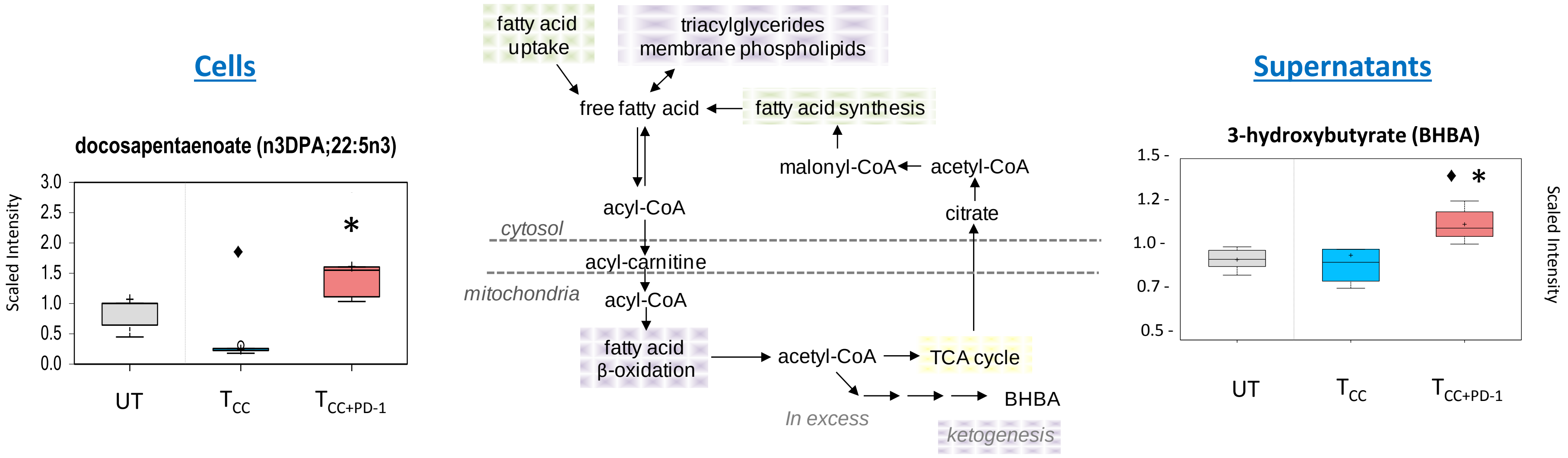

Supplementary figure 3. PD-1 inhibits lipid biosynthesis and promotes fatty oxidation during T cell activation. The amounts of n3DPA;22:5n3 and 3-hyroxybutyrate (BHBA) were analyzed under the indicated culture conditions and values were plotted in whisker boxes. A schematic outline of fatty acid metabolism is shown in parallel.

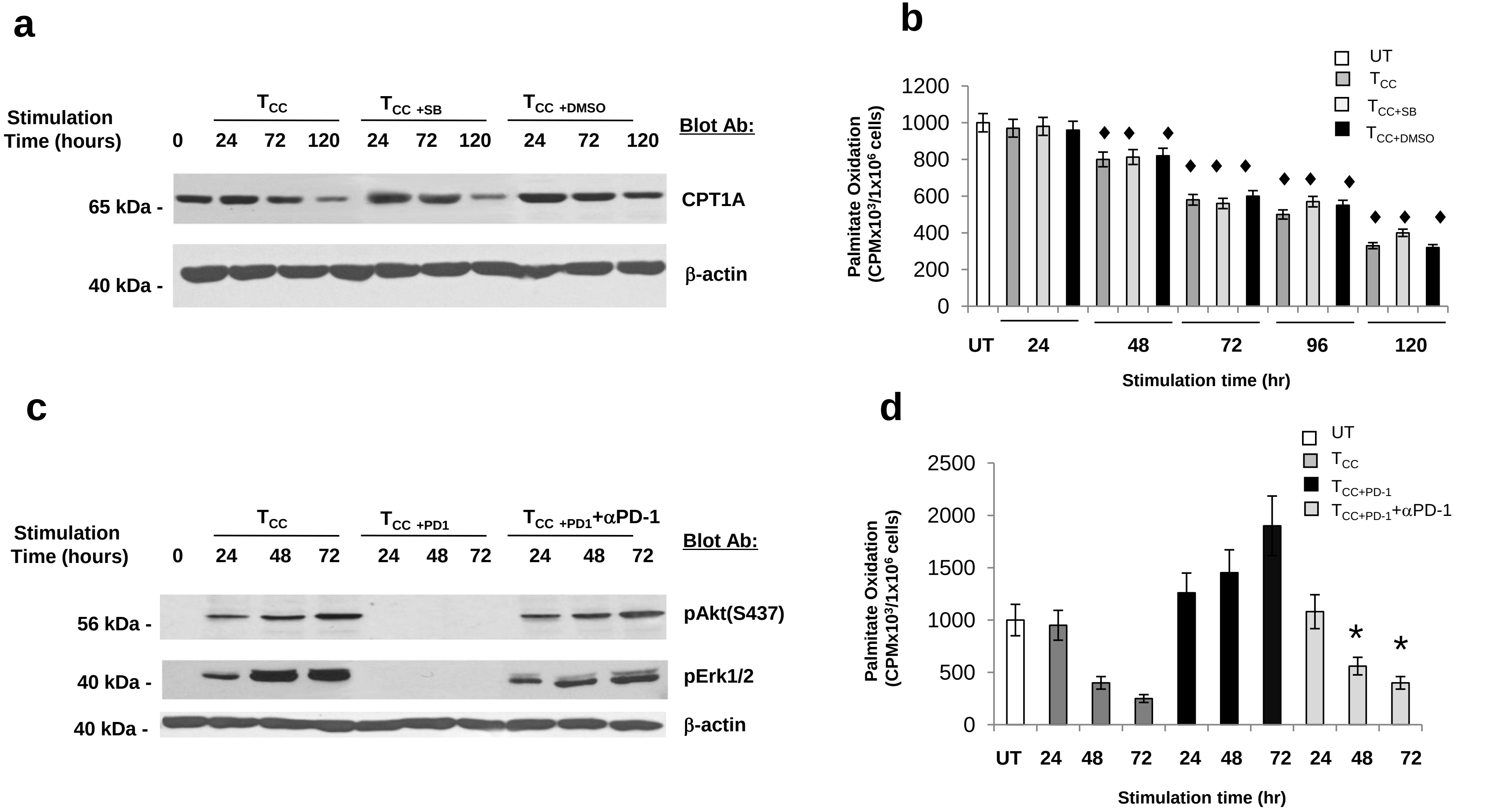

Supplementary figure 4. (A-B) Vehicle control and the p38 MAPK specific inhibitor SB203580 do not affect CPT1A expression or FAO upon T cell activation. (A) CD4⁺ human T cells were cultured with tosylatcivated magnetic beads conjugated with aCD3/aCD28/IgG (T_{CC}) in the presence of either SB203580 (10 uM) or vehicle control (DMSO). Cell lysates were prepared at the indicated time points and expression of CPT1A and β-actin was assessed by SDS-PAGE and immunoblot. Results are representative of two experiments. **(B)** In parallel experiments, fatty acid β-oxidation rate was examined. Values of T_{CC}, T_{CC}+SB and T_{CC}+DMSO cultures were compared to unstimulated (UT) (♦$P<0.05$). **(C-D)** Blockade of PD-1 restores Akt and Erk1/2 activation and results in reduced rate of FAO. CD4⁺ primary human T cells were either left unstimulated (UT) or were incubated with magnetic beads conjugated with aCD3/aCD28/PD-L1-Ig (T cells costimulated+PD-1; T_{CC+PD1}) alone or in the presence of an anti-PD-1 blocking antibody (J105; eBioscience). Activation of Akt and Erk1/2 was examined with phospho-specific antibodies. Fatty acid β-oxidation rate after culture under the indicated conditions was examined. Results are representative of two experiments.

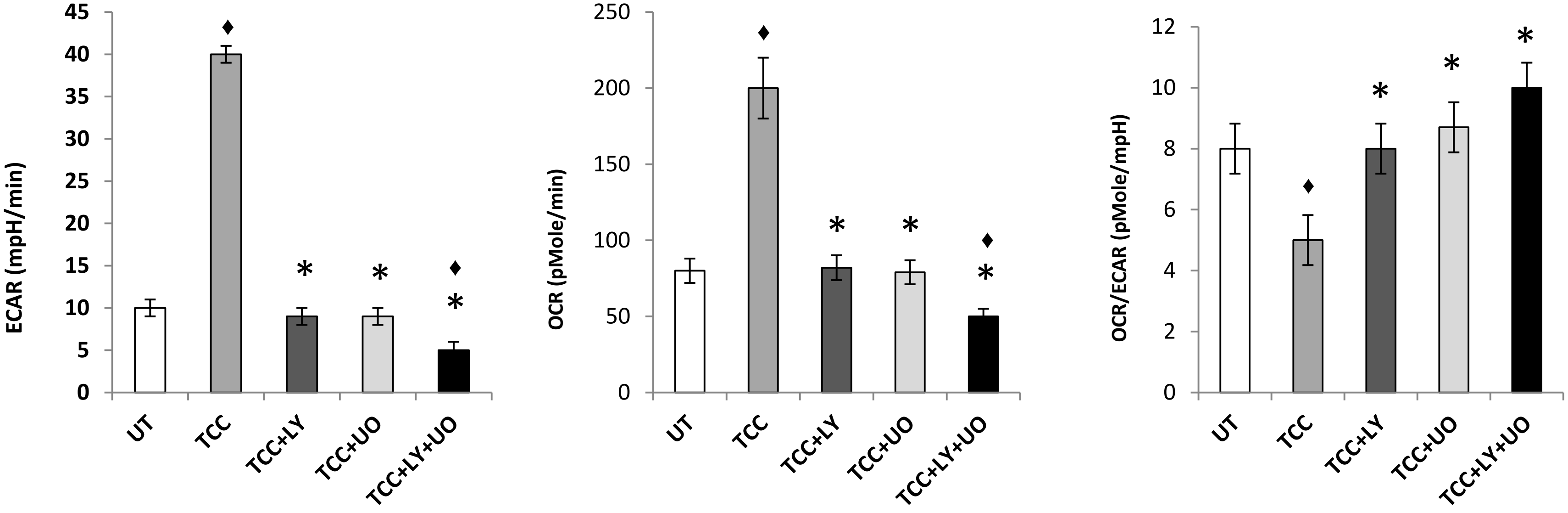

Supplementary figure 5. Effects of LY294002 and UO126 on the bioenergetics of activated T cells. $CD4^+$ primary human T cells were cultured with tosylactivated magnetic beads conjugated with anti-CD2/CD28/IgG (T_{CC}) alone or in the presence of LY294002 (LY), UO126 (UO) or their combination. At 72 hours of culture, extracellular acidification rate (ECAR) and oxygen consumption rates (OCR) were assessed. **(B)** OCR/ECAR ratio was also determined. Values of each experimental conditions were compared to unstimulated (UT) cells (♦P<0.05, n = 3) and values in T_{CC}+inhibitor cells were compared to T_{CC} (*P<0.05, n = 3).

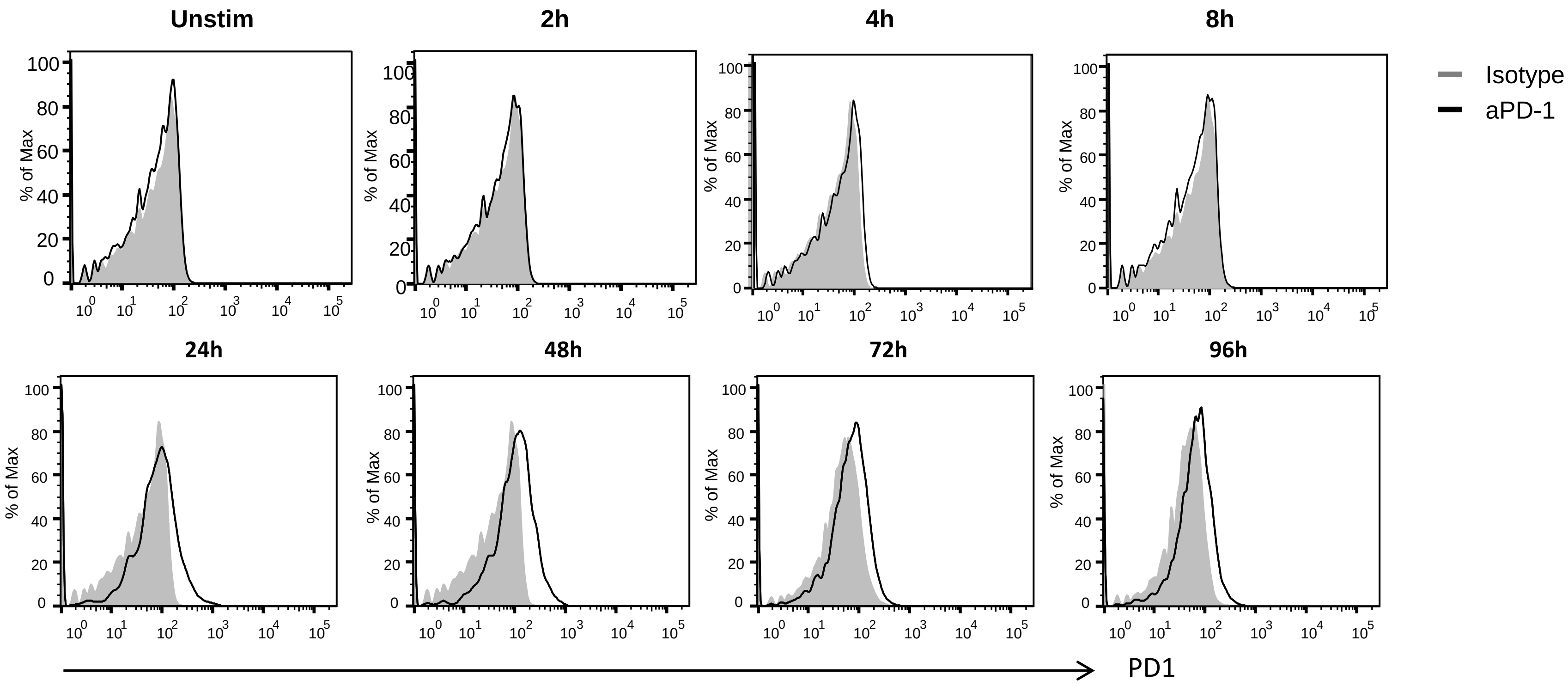

Supplementary figure 6. Detectable PD-1 expression on the cell surface is not rapidly induced upon T cell activation.
CD4$^+$ primary human T cells were cultured with anti-CD3-plus-anti-CD28 mAbs and expression of PD-1 at the indicated time points was examined by flow cytometry. Results are representative of three independent experiments.

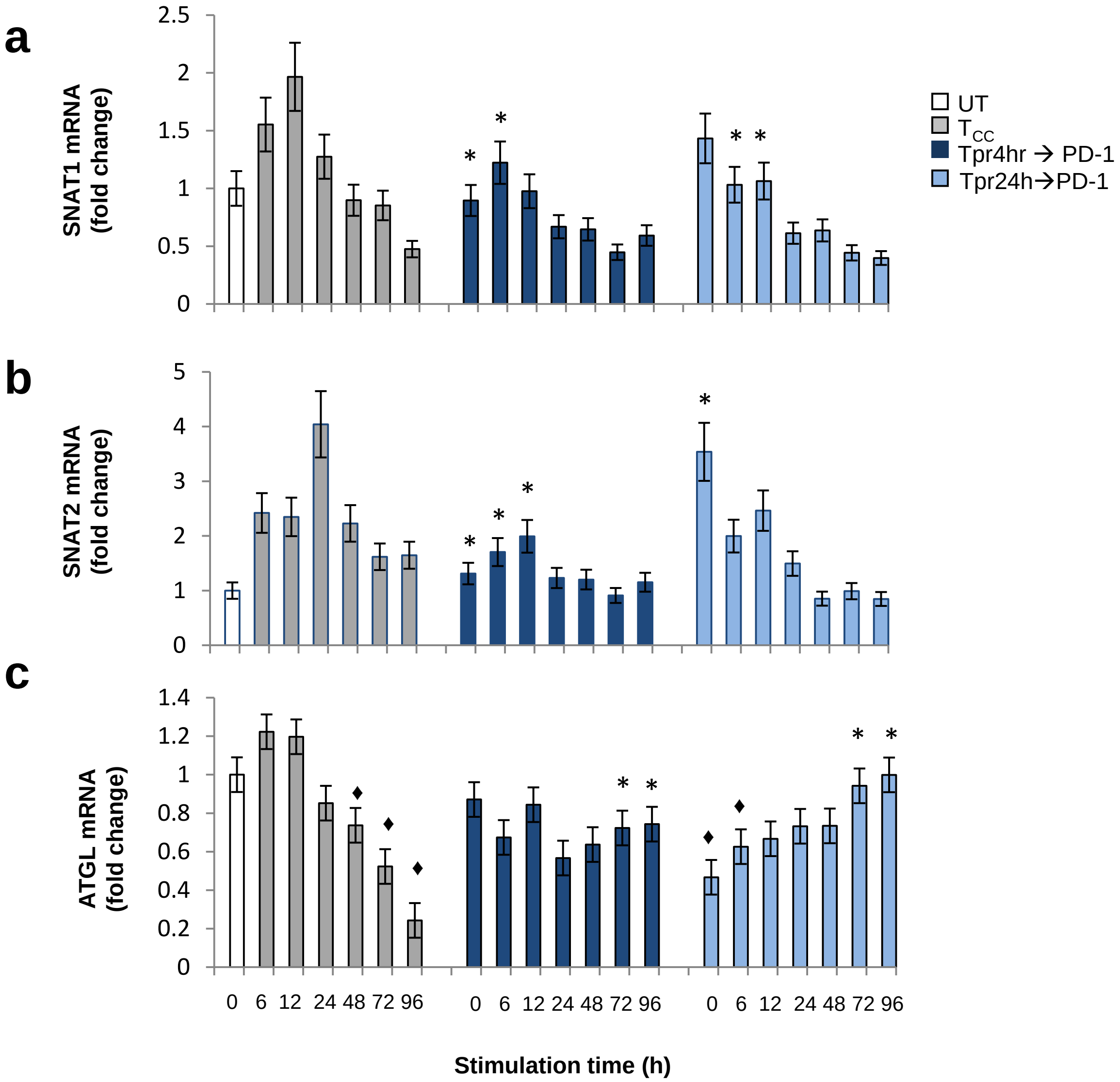

Supplementary figure 7. **PD-1 suppresses the expression of SNAT1 and SNAT2 and upregulates the expression of ATGL in pre-activated human T cells**. $CD4^+$ T cells were cultured with anti-CD3-and-anti-CD28 mAbs for 4 hours or for 24 hours and subsequently were collected, rested for three hours and re-cultured with tosyl-activated magnetic beads (1.5 x 10^5 beads / well) conjugated with anti-CD3, anti-CD28 mAbs and PD-L1-IgG2a. In the same experiment $CD4^+$ T cells stimulated with tosyl-activated magnetic beads (1.5 x 10^5 beads/well) conjugated with anti-CD3, anti-CD28 mAbs and IgG2a were used as positive control.
